# Dual targeting of HER1/EGFR and HER2 with cetuximab and trastuzumab in patients with metastatic pancreatic cancer after gemcitabine failure: results of the “THERAPY”phase 1-2 trial

**DOI:** 10.18632/oncotarget.3473

**Published:** 2015-02-28

**Authors:** Eric Assenat, David Azria, Caroline Mollevi, Rosine Guimbaud, Nicole Tubiana-Mathieu, Denis Smith, Jean-Pierre Delord, Emmanuelle Samalin, Fabienne Portales, Christel Larbouret, Bruno Robert, Frédéric Bibeau, Jean-Pierre Bleuse, Evelyne Crapez, Marc Ychou, André Pèlegrin

**Affiliations:** ^1^ Centre Hospitalier Régional Universitaire (CHU) de Montpellier, Montpellier, France; ^2^ Institut Régional du Cancer de Montpellier (ICM)-Val d'Aurelle, Montpellier, France; ^3^ IRCM, Institut de Recherche en Cancérologie de Montpellier, Montpellier, France; ^4^ INSERM, U896, Montpellier, France; ^5^ Université Montpellier 1, Montpellier, France; ^6^ Institut Régional du Cancer de Montpellier, ICM, Montpellier, France; ^7^ Centre Hospitalier Universitaire (CHU) de Toulouse, TSA, Toulouse cedex, France; ^8^ Centre Hospitalier Universitaire (CHU) de Limoges, Limoges cedex, France; ^9^ Centre Hospitalier Universitaire (CHU) de Bordeaux, Talence cedex, France; ^10^ Institut Claudius Régaud, Toulouse, France

**Keywords:** pancreatic cancer, cetuximab, trastuzumab, phase 1/2, antibody combination

## Abstract

To improve treatment efficacy, we decided to simultaneously target HER1 and HER2 with trastuzumab and cetuximab. Following promising preclinical results, we conducted a phase 1-2 trial in advanced pancreatic cancer patients after first-line gemcitabine-based chemotherapy failure. In this single-arm, non-randomized, multicenter trial, patients received weekly cetuximab (400mg/m², then 250mg/m²). They were sequentially included in two trastuzumab dose levels: 3.0 or 4.0mg/kg, then 1.5 or 2.0mg/kg/weekly. Endpoints were the objective response rate, safety, progression-free (PFS) and overall survival (OS). During phase 1 (n=10 patients), toxicities were evenly distributed except for skin toxicities that frequently caused compliance issues. The higher dose level was defined as the trastuzumab recommended dose. During phase 2 (n=39 patients), toxicities were mainly cutaneous reactions and asthenia. No objective response was observed. Nine patients were stabilized but arrested treatment due to toxicity. Median PFS was 1.8 months (95%CI: 1.7-2.0 months) and median OS was 4.6 months (95%CI: 2.7–6.6 months). Both were positively correlated with skin toxicity severity (P=0.027 and P=0.001, respectively). Conventional phase 1 dose-escalation schedules are unsuitable for targeted therapies because most cutaneous toxicities are not considered dose-limiting toxicities. The compliance issues caused by skin toxicities were particularly detrimental because of the toxicity-response correlation.

## INTRODUCTION

Pancreatic cancer is the tenth most common cause of cancer in the United States and the fourth leading cause of cancer death, with an estimated 42 000 new cases and 35 000 associated deaths in 2009 [1]. Despite its modest clinical benefit, gemcitabine has been the only approved first-line treatment for advanced pancreatic cancer for 15 years [2]. Combinations of fluorouracil, irinotecan, oxaliplatin and leucovorin (FOLFIRINOX), or gemcitabine and nab-paclitaxel are often administered as first-line treatment to patients with metastatic pancreatic cancer and a good performance status [3, 4]. To increase the chemotherapy efficacy, several studies have assessed the combination of gemcitabine with targeted therapies; however, most regimens evaluated in phase 3 clinical trials failed to show any overall survival (OS) improvement [5-9]. Indeed, only one randomized trial [8] (*n=*569 patients) that compared gemcitabine alone or combined with erlotinib showed a modest, but significant OS increase in the erlotinib+ gemcitabine arm (5.9 vs 6.2 months, *P=*0.025).

The expression and role of epidermal growth factor receptors (EGFR/HER1 and HER2) have been widely studied, including in pancreatic cancer [10-15]. Moreover, 17% to 33% of pancreatic adenocarcinomas overexpress HER2, and HER2-positive status has also been correlated with shorter survival [10]. With the aim of optimizing therapeutic strategies, EGFR expression in pancreatic tumors has been determined in some clinical trials in order to assess its potential role in predicting treatment efficacy.

As part of a pilot study, the combination of trastuzumab (TRA; anti-HER2 monoclonal antibody) and gemcitabine was well tolerated, but showed limited therapeutic benefit in 34 patients with HER2-overexpressing metastatic pancreatic cancer [16]. Cetuximab (CET; anti-HER1 monoclonal antibody) was also assessed in combination with gemcitabine in a phase 2 study that included 41 patients with advanced pancreatic cancer overexpressing HER1 [17]. However, the results of two recent randomized studies combining gemcitabine-based chemotherapy with CET were disappointing [9, 18].

We therefore decided to assess a strategy in which HER1 and HER2 are simultaneously targeted. In a first preclinical study, we found that the trastuzumab + matuzumab (anti-HER1 antibody) combination was more efficient in inhibiting tumor progression in mice xenografted with human pancreatic carcinoma cell lines than each antibody alone. This synergistic effect was associated with a decrease in HER1/HER2 phosphorylation [19]. We then observed that the TRA + CET combination was more efficient as first- and second-line treatment than the standard chemotherapy (gemcitabine) in nude mice bearing human pancreatic cancer xenografts [20]. Finally, we demonstrated that the TRA + CET combination had a better synergistic effect than TRA + erlotinib or lapatinib alone in xenografted mice [21].

Here, we report the results of the first multicenter phase 1-2 clinical trial to evaluate the TRA + CET combination for the treatment of patients with advanced pancreatic cancer after failure of gemcitabine-based first-line chemotherapy.

## RESULTS

**Figure 1:**
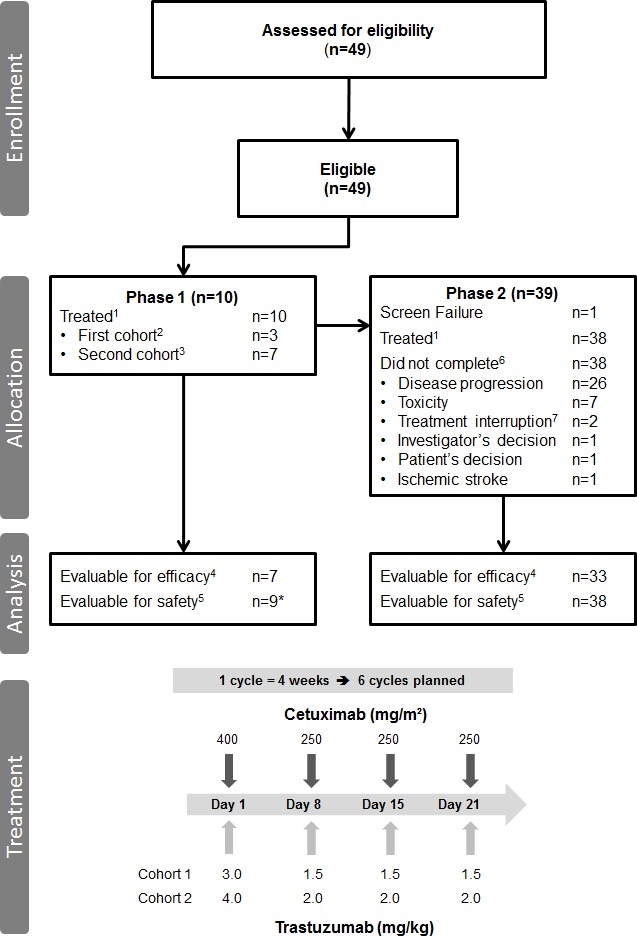
Study flow diagram and administration schedules ^1^Patients who received at least one dose of treatment. ^2^Trastuzumab: 3.0 mg/kg on day 1 and then 1.5 mg/kg on day 8, day 15 and day 22. ^3^Trastuzumab: 4.0 mg/kg on day 1 and then 2.0 mg/kg on day 8, day 15 and day 22. ^4^Patient who completed at least two cycles of treatment or discontinued due to disease progression. ^5^Patient who received at least one dose of treatment and with available data. ^6^Treatment completion: six cycles of treatment. ^7^Delay > 15 days. *One patient (cohort 2) received only the first injection after two lines of chemotherapy (gemcitabine in adjuvant setting and gemcitabine-oxaliplatin as first-line metastatic chemotherapy). The patient died because of acute digestive obstruction (hepatic and peritoneal metastasis) before safety evaluation.

Over a 15-month period, 10 patients were enrolled in the phase 1 and 39 patients in the phase 2 of this study (Fig. 1). One patient could not be evaluated in phase 2 because of screening failure. Table 2 summarizes the baseline characteristics of the enrolled patients. All patients had previously received gemcitabine-based chemotherapy and many patients had also been treated with a second-line or even later cycles of palliative chemotherapy; 60% (phase 1) and 79% (phase 2) of patients had liver metastases and half of our study population had serum Ca 19.9 ≥ 65 UI/mL (without jaundice). Safety could not be evaluated in two patients (Fig. 1).

**Table 1:** THERAPY clinical trial: eligibility criteria

**Inclusion criteria**
previous failure of gemcitabine-based chemotherapy (in adjuvant or metastatic settings)
measurable lesion according to RECIST version 1.0
WHO performance status ≤ 1
age ≥ 18 years
life expectancy ≥ 3 months
left ventricular ejection fraction ≥ 55%
adequate organ function: absolute neutrophil count ≥ 1500 cells per μL hemoglobin > 9 g/dL platelet count ≥ 100 000 cells per μL creatinine function < 1.5 × ULN serum bilirubin ≤ 2.5 × ULN serum transaminases ≤ 5 × ULN)
**Exclusion criteria**
brain metastases or symptomatic leptomeningeal carcinomatosis
other concurrent cancer (except for skin basal cell carcinoma)
prior chemotherapy with CET or TRA
hypersensitivity to CET or TRA
pregnancy or lactation
fertile patient (man or woman) without effective contraception
concomitant treatment with other experimental drugs or any other anticancer therapy
significant comorbidities such as: cardiovascular disease (documented congestive cardiac failure, high risk unstable arrhythmia, angina pectoris requiring treatment, significant valvulopathy, sign of myocardial infarction or unstable high arterial blood pressure), active bleeding, clinically significant active infection, or severe or oxygen-dependent dyspnea at rest
Abbreviations: CET, cetuximab; RECIST, Response Evaluation Criteria in Solid Tumors; TRA, trastuzumab; ULN, upper limit of normal

**Table 2:** Demographics and baseline characteristics

	Phase 1 n=10		Phase 2 n=38	
Age, years				
Median	58.5		63.0	
Range	41-61		43-78	
Sex				
Male	5	(50%)	25	(66%)
Female	5	(50%)	13	(34%)
WHO performance status (PS)				
0	3	(30%)	24	(63%)
1	6	(60%)	13	(34%)
2	1*	(10%)	1*	(3%)
Location of primary tumor				
Head of pancreas	5	(50%)	23	(60%)
Tail of pancreas	2	(20%)	8	(21%)
Body of pancreas	3	(30%)	7	(19%)
Primary tumor surgery	4	(40%)	16	(42%)
Primary tumor radiotherapy	2	(20%)	8	(21%)
Previous chemotherapy treatment**				
Adjuvant chemotherapy	2	(20%)	16	(42%)
with gemcitabine	2	(100%)	15	(94%)
with fluorouracil	0	-	1	(6%)
Palliative chemotherapy	10	(100%)	32	(84%)
with gemcitabine	9	(90%)	20	(62%)
FOLFIRINOX	1	(10%)	0	-
1^st^ line	4	(40%)	19	(59%)
≥ 2^nd^ line	6	(60%)	13	(41%)
Metastatic disease - Number of sites				
1	9	(90%)	25	(66%)
2	1	(10%)	9	(24%)
> 2			4	(10%)
Serum Ca 19.9				
< 65 UI/mL	3	(50%)	14	(52%)
≥ 65 UI/mL	3	(50%)	13	(48%)
Missing			11	

Abbreviations: WHO, World Health Organization

* Enrolled patient although not meeting the inclusion criterion of PS ≤ 1

** All patients received gemcitabine (adjuvant or palliative chemotherapy)

In the phase 1 part, patients in cohort 1 (*n=*3) received two cycles of treatment and patients in cohort 2 (*n=*7) received one or two cycles of treatment (but for one patient who received six cycles) (Fig. 1). The median duration of treatment was 1.9 months (range: 0.9-5.8 months). In cohort 1 (*n=*3), the median relative dose intensity (RDI) was 89% for both CET and TRA. In cohort 2 (*n=*7), the median RDI was 65% for CET and 67% for TRA. Indeed, toxicities (not considered as DLTs) occurred in all patients and were responsible for treatment delay or interruption. Premature discontinuation was due to disease progression in nine patients and to skin toxicity in one patient from cohort 2. This toxicity event required a treatment delay longer than 15 days and was thus considered as a DLT (the only one in the phase 1 of this study). Two patients died from disease-related events. The main toxicities included asthenia (grade 1: one event; grade 2: four; grade 4: two), anorexia (grade 1: one event; grade 2: two; grade 3: one) and rash/acne (grade 1: two events; grade 3: five). Grade 3 toxicities were evenly distributed, except for skin toxicities that were more frequent (two patients in cohort 1, and three patients in cohort 2). Based on these results, the recommended doses for the phase 2 study were 400 mg/m² CET (loading dose) followed by a 250 mg/m² dose, combined with 4.0 mg/m² TRA (loading dose) and then 2.0 mg/kg.

In the phase 2 study, 10 patients (26%) received one cycle, 21 (55%) received two cycles and 7 patients (19%) received three to five cycles of treatment. The median treatment duration was 1.8 months (range: 0.9-4.8 months). The median RDI was 67% for CET and 74% for TRA (range: 0% - 100% for both). A RDI ≥ 80% was obtained in 12 patients (32%) for CET and in 13 patients (34%) for TRA. Eighteen patients (47%) prematurely stopped the treatment mainly due to toxicity or disease progression (two patients died from disease-related events). Phase 2 toxicities are detailed in Table 3. No toxicity-related death was reported and most adverse events were grade 1 or 2. The most commonly reported grade 3 adverse events were cutaneous toxicities (*n=*19). Other non-cutaneous grade 3 toxicities included asthenia (*n=*6), metabolism alterations (*n=*4), hematological (*n=*4) and cardiovascular (*n=*4) events. The three reported grade 4 toxicities were thrombosis/embolism (*n*=2) and asthenia (*n*=1, attributed to the disease).

**Table 3:** Phase 2 toxicities (n=38)

	Grade 1-2		Grade 3	
Cutaneous				
Rash / Acne	18	(47%)	12	(32%)
Fissures	11	(29%)	3	(8%)
Pruritus	11	(29%)	2	(5%)
Skin dryness	20	(53%)	1	(3%)
Paronychia	9	(24%)	1	(3%)
Nail toxicity	2	(5%)	-	-
Urticaria	1	(3%)	-	-
Hematological				
Neutropenia	3	(8%)	-	-
Lymphopenia	3	(8%)	2	(5%)
Anemia	20	(52%)	1	(3%)
Thrombocytopenia	9	(24%)	1	(3%)
Metabolism				
Hyperbilirubinemia	-	-	3	(8%)
Hypokalemia	3	(8%)	1	(3%)
Gastrointestinal				
Mucositis / Stomatitis	17	(44%)	3	(8%)
Anorexia	18	(47%)	1	(3%)
Diarrhea	8	(21%)	1	(3%)
Abdominal Pain	17	(44%)	1	(3%)
Vomiting	7	(18%)	1	(3%)
Cardiovascular				
Thrombosis / Embolism	1	(3%)	5*	(13%)
Arterial hypertension	-	-	1	(3%)
Other				
Asthenia	19	(50%)	7**	(18%)
Allergy / Hypersensitivity	1	(3%)	2	(5%)
Edema	3	(8%)	1	(3%)

* including two grade 4 events;

** including one grade 4 event

Efficacy could be evaluated only in 33 patients (Fig. 1) because six patients prematurely withdrew from the study (not because of disease progression). The phase 2 median follow-up was 3.8 months (range: 0.4-22.9 months). No objective response was observed. The responses relative to the severity of the cutaneous toxicities are presented in Table 4A. Nine patients were stabilized, but discontinued the treatment due to toxicity.

**Table 4A:** Response in phase 2 patients relative to the cutaneous toxicity grade

Phase 2 (n=33)	Stable Disease	Progressive Disease	*P*-value1
All evaluable patients	9 (27%)	24 (73%)	
With grade 0-1 skin toxicities*	-	8 (33%)	0.047
With grade ≥ 2 skin toxicities*	9 (100%)	16 (67%)	
With grade 0-1 skin toxicities**	-	10 (42%)	0.020
With grade ≥ 2 skin toxicities**	9 (100%)	14 (58%)	

1correlation between toxicity and response to treatment

* Including all cutaneous toxicities

** Including pruritus, rash/acne, skin dryness, urticaria and paronychia

**Figure 2:**
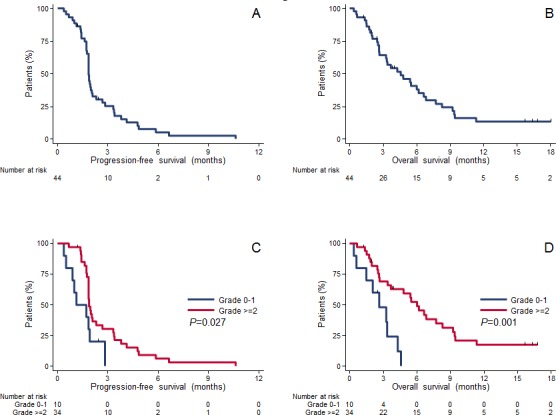
Kaplan Meier progression-free survival and overall survival curves of all patients, and progression-free survival and overall survival curves of patients who experienced grade 0-1 or grade ≥ 2 cutaneous toxicities (all types)

For the survival analysis, the data of all evaluable patients included in the phase 2 study (n=38) were pooled with those of the phase 1 patients who received the same TRA dose (cohort 2, n=6). The median follow-up for the pooled patients (n=44) was 3.7 months (range: 0.4-22.9 months). The median PFS was 1.8 months (95% CI: 1.7 - 2.0 months), while the median OS was 4.6 months (95% CI: 2.7 – 6.6 months) (Fig. 2A and 2B). Both PFS and OS were significantly (and positively) correlated with the severity of skin toxicities (Fig. 2C and 2D), as detailed in Table 4B.

**Table 4B:** Survival in phase 1 + 2 patients relative to the cutaneous toxicity grade

Phase 1 + 2 (n=44)	PFS Months (95% CI)	OS Months (95% CI)	*P*-value1	
			PFS	OS
All evaluable patients	1.8 (1.7-2.0)	4.6 (2.7-6.6)		
with grade 0-1 skin toxicities*	1.1 (0.4-1.9)	2.6 (0.4-4.3)	0.027	0.001
with grade ≥ 2 skin toxicities*	1.9 (1.8-2.3)	6.0 (3.4-8.3)		
with grade 0-1 skin toxicities**	1.7 (0.6-1.8)	3.3 (0.7-4.6)	0.006	0.002
with grade ≥ 2 skin toxicities**	1.9 (1.8-3.3)	6.2 (2.7-9.2)		

Abbreviations: PFS, progression-free survival; OS, overall survival

1correlation between toxicity and survival, log-rank test

* Including all cutaneous toxicities

** Including pruritus, rash/acne, skin dryness, urticaria and paronychia

Concerning the ancillary experiments, KRAS status could be assessed in 24/44 patients (55%) and HER1 and HER2 expression levels in 25/44 patients (57%) and 28/44 patients (64%), respectively (Table 5). Fcγ receptor polymorphisms (Fc?RIIA-H131R and FcγRIIIA-V158F) were determined in 29/44 patients (66%). Four tumors (16%) expressed both HER1 and HER2 and in three of them a KRAS mutation was detected. No statistical correlation was found between these five parameters which can be involved in the efficacy of CET [25], TRA [26] or mAbs in general [25], and the response to treatment or cutaneous toxicity.

**Table 5:** KRAS mutational status, HER1/EGFR and HER2 expression and Fc γ receptor polymorphisms (n=44 tumors)

	n (%)	
KRAS		
Wild type	9	(38%)
Mutated	15	(62%)
Missing	20	
HER1		
Negative	10	(40%)
Positive	15	(60%)
Missing	19	
HER2		
Negative	20	(71%)
Positive	8	(29%)
Missing	16	
FcγRIIA-H131R		
H/H	8	(28%)
H/R	19	(65%)
R/R	2	(7%)
Missing	15	
FcγRIIIA-V158F		
F/F	13	(45%)
V/F	14	(48%)
V/V	2	(7%)
Missing	15	

## DISCUSSION

This phase 1-2 trial confirms the feasibility of combining cetuximab and trastuzumab for the treatment of patients with metastatic pancreatic adenocarcinoma after first-line chemotherapy failure. However, the primary objective of a 5% to 20% response rate was not achieved because no complete or partial response could be observed, but only prolonged stable disease in nine patients with grade 3-4 cutaneous toxicities. Although the results of this trial are negative based on its initial design, several lessons can be drawn from our observations.

First, the absence of complete or partial response could lead to question the anticancer activity of combined CET-TRA. Indeed, antibodies targeting HER1 or HER2 are not efficient when administered individually with conventional chemotherapy in advanced pancreatic cancer [9, 16, 18]. However, our survival results should be compared with those obtained with second-line palliative chemotherapy after gemcitabine failure. In our study, 23 patients (48%) had already received palliative chemotherapy as first-line treatment and 19 patients (40%) had already received palliative chemotherapy as second-line or even later treatment. In a randomized phase 3 trial that compared best supportive care (BSC) with combined oxaliplatin/folinic acid/5-FU (OFF regimen) as second-line treatment for patients with advanced pancreatic cancer, OS was 4.82 months [95% CI: 4.29-5.35] in the OFF arm and 2.30 months [95% CI: 1.76-2.83] in the BSC arm (*P=*0.008) [27]. In another phase 3 randomized trial evaluating cisplatin/folinic acid/5-FU *vs* gemcitabine as first- and second-line treatment for patients with metastatic pancreatic cancer [28], PFS (from second-line start to progression) was 1.6 months, which is similar to our results. Moreover, patients in which disease was stabilized discontinued the treatment due to toxicity, suggesting that more work should be done to determine the optimal dose to allow good efficacy with acceptable toxicities.

Indeed, the way the recommended dose was determined in the phase 1 trial should be discussed. The traditional DLT definition, which focuses on grade 3-4 toxicities occurring only during the first treatment cycle, was designed for conventional chemotherapy. Therefore, it may not be appropriate for non-cytotoxic agents for which late, different or lower grade toxicities also deserve attention [29]. As recently highlighted by Paoletti et al. [30], DLT assessment should take into account also lower grade toxicities that lead to significant RDI decrease and that may also occur after the first treatment cycle. The design of phase 1 trials to assess non-cytotoxic agents should allow for precise dose adjustments to achieve > 75% RDI. In our study, the 3+3 standard escalation scheme was clearly inappropriate because many grade 3 cutaneous toxicities occurred during the phase 1 part (> 50% of patients). These events led to a significant RDI decrease for both targeted therapies, but could not be considered as DLTs. These compliance issues were confirmed by the phase 2 study. Specifically, nine patients discontinued the treatment due to cutaneous toxicity, although disease was stabilized. Moreover, such cutaneous adverse events might be more frequent and severe in patients who are concomitantly treated with two targeted therapies to block both HER1 and HER2 [31, 32]. Therefore, extra care should be taken when moving from preclinical to clinical settings, for example by starting with lower doses than those used when these compounds are used individually.

Finally, the correlation between response or survival and severity of cutaneous adverse events, as observed in our study, has already been reported. For instance, the erlotinib-gemcitabine combination, which showed some efficacy improvement compared to the individual drugs in patients with pancreatic cancer, was associated with increased toxicity [8]. In a retrospective study, 168 patients with pancreatic cancer and treated with combined erlotinib and gemcitabine were classified in two groups (high and low severity), based on the rash intensity [33]. The high severity group had longer median OS and PFS than the low severity group (both *P<*0.05); patients suffering from particularly severe rash also had a lower risk of death (hazard ratio [HR] 0.67, *P<*0.05). Likewise, in a phase 2 trial that enrolled patients with metastatic pancreatic cancer (*n=*64) treated with chemotherapy + CET, the presence of a rash was significantly correlated with OS [34]. Moreover, as this efficacy-toxicity association had been described also in patients with colorectal cancer (CRC), Van Cutsem et al. (34) conducted the EVEREST trial in 157 patients who experienced grade ≤ 1 skin reactions following standard-dose CET. These patients were randomly assigned to receive weekly standard-dose or dose-escalated CET (500 mg/m²) [35]. This dose escalation was associated with increased skin toxicity, but also with improved response rate and disease control rate. A second trial conducted by the same group is in progress to determine whether administering escalating doses of cetuximab in patients with no early skin toxicity could delay the progression of the disease in a significant proportion of patients and to study the molecular signatures of response (NCT01251536). Similarly, in a phase 2 randomized trial in patients with metastatic CRC treated with CET, occurrence of grade 2-3 skin toxicities (compared with grade 0-1) was correlated with improved outcome, including better overall response rate, PFS and OS [36]. The authors also highlighted the independence of this effect from the *KRAS* mutation status and suggested that patients' constitutional factors were possibly involved in the relationship between response to CET and skin toxicity. Concerning the underlying mechanism, as EGFR is involved in the maintenance of epithelial homeostasis, EGFR inhibitors will target also normal epithelial cells. Takata et al. [37] suggested that they might particularly affect cell differentiation of epidermal keratinocytes and sebaceous glands. Moreover, EGFR and HER2 dimerization status could explain the different type of toxicity induced by EGFR-targeted *vs* HER2-targeted therapies [38]. Finally, another study assessed the safety and efficacy of everolimus, an inhibitor of mTOR, which is downstream of HER1 and HER2 [39]. However, because of toxicity (mainly cutaneous), the recommended dose could not be determined in this phase 1-2 trial. In view of all these data, future clinical investigations need to include both cutaneous and tumor biopsies as they might help understanding whether and how the mechanism of action and efficacy of EGFR targeted therapies is linked to dose-limiting cutaneous reactions.

In conclusion, although this phase 1-2 trial did not bring the expected efficacy results for the treatment of advanced pancreatic cancer, one can learn from our observations. Conventional phase 1 dose-escalation schedules are clearly not appropriate for targeted therapy evaluation as most of the induced cutaneous toxicities are not considered as DLTs. However, they often entail dose reduction or treatment interruption, which is particularly detrimental because such toxicities have been positively correlated with response to treatment.

### Statement of translational relevance

As HER family members are involved in pancreatic carcinoma and are activated through dimerization, we tested the combination of cetuximab (anti-EGFR/HER1) and trastuzumab (anti-HER2 antibody). Following promising preclinical results, the aim of this phase 1-2 trial was to determine the trastuzumab recommended dose and to assess the combination safety and efficacy. Nine patients out of 39 were stabilized, but stopped the treatment because of toxicity. The frequency and severity of cutaneous toxicities were correlated with survival, but also caused treatment compliance issues. Our study demonstrates that the conventional phase 1 design and the associated definition of dose-limiting toxicities are inappropriate for the evaluation of targeted therapy-based regimens, especially when directed against HER1/2. Cutaneous toxicities, which are not considered as dose-limiting, should be taken into account for future dose-escalation studies to evaluate the potential of anti-HER antibody combinations. Investigation of the underlying mechanisms might help understanding this significant toxicity-efficacy correlation.

## PATIENTS AND METHODS

### Study design and patients

We designed a phase 1-2, open-label, single-arm, non-randomized, multicenter trial. The study protocol was approved by our local ethics committee, the French competent authorities, and our Institutional Gastrointestinal Review Board. The study was conducted in accordance with the Declaration of Helsinki and Good Clinical Practice. Written informed consent has been obtained from all patients before entering the study. The phase 1 study was only performed in Montpellier, while seven French centers participated in the phase 2 trial. Patients with histologically documented, unresectable pancreatic adenocarcinoma were enrolled (Fig. 1). The other eligibility criteria are listed in Table 1.

### Phase 1 trial

The phase 1 trial included two consecutive cohorts corresponding to the two TRA dose levels (Fig. 1). Both cohorts received weekly intravenous CET at a loading dose of 400 mg/m² (day 1, week 1, 90-minute infusion) and then at 250 mg/m² (30-minute infusion). Dose escalation was provided for TRA only, which was administered 1 hour after the end of the CET infusion. A standard 3+3 dose escalation design with three to six patients per dose level was used. Patients in the first cohort received 3.0 mg/kg TRA as loading dose (day 1, week 1, 90-minute infusion) followed by 1.5 mg/kg TRA the following weeks (30-minute infusion). If the tolerance was acceptable in this first cohort, the second cohort (second dose level) received 4.0 mg/kg TRA (loading dose) the first week and then 2.0 mg/kg TRA. A treatment cycle was defined as four weeks of treatment. The phase 1 primary endpoint was the recommended dose of TRA defined as the dose for which one type of dose-limiting toxicity (DLT) occurred at most in 33% of patients during the first two treatment cycles. Adverse events were graded according to the National Cancer Institute Common Terminology Criteria for Adverse Events (CTCAE), version 3.0. DLT was defined as any grade 4 toxicity (except for alopecia, or vomiting in the absence of adequate prophylaxis), fever or sepsis concurrent with grade 3-4 neutropenia, symptomatic thrombocytopenia (hemorrhage), any grade 3 cardiotoxicity and any toxicity requiring a treatment delay longer than 15 days. Treatment was to be administered for six complete cycles or until disease progression, patient refusal or unacceptable toxicity.

### Phase 2 trial

After completion of the phase 1 part, the phase 2 trial assessed the CET and TRA combination according to the recommended dose defined based on the phase 1 results. Adverse events were managed as in phase 1. The phase 2 primary endpoint was the objective response rate (ORR). Every four weeks, target lesions were assessed by independent review of thorax-abdomen-pelvis computed tomography scans according to Response Evaluation Criteria in Solid Tumors (RECIST), version 1.0 [22]. The tumor response had to be confirmed by two consecutive assessments separated by a four-week interval. Secondary endpoints were the tolerance profile, progression-free survival (PFS) and OS. Routine laboratory tests were done weekly during the first two cycles and every two weeks afterwards; physical examination was performed before each treatment.

### Ancillary study

An optional ancillary study assessed the predictive value of individual biomarkers, such as *KRAS* mutations, HER1 and HER2 expression, and Fcγ receptor polymorphisms (FcγRIIA-H131R and FcγRIIIA-V158F). Expression of HER1 and HER2 in surgical specimens was evaluated by immunohistochemistry, as previously described [23]. *KRAS* mutations in tumor tissues were determined by high resolution melt analysis and direct sequencing [24]. Polymorphism genotyping was performed, as previously described [25].

### Statistical methods

Three to 12 patients (3 to 6 per cohort) were planned for the phase 1 study. For the phase 2 study, 24 to 55 patients were required assuming a Simon's optimal two-stage design with α=5% and β=10%. The minimal ORR value was estimated at 5% and treatment was considered promising if ORR ≥ 20%.

PFS was calculated from inclusion until disease progression or death. Patients alive without progression were censored at the time of the last contact. OS was calculated from inclusion until death. Patients alive or lost to follow-up were censored at the time of the last contact. The Kaplan-Meier method was used to estimate PFS and OS. A p-value of 0.05 was considered as statistically significant.

This study was registered at ClinicalTrials.gov, number NCT00923299.
